# Predicting the Evolution of Physics Research from a Complex Network Perspective

**DOI:** 10.3390/e21121152

**Published:** 2019-11-26

**Authors:** Wenyuan Liu, Stanisław Saganowski, Przemysław Kazienko, Siew Ann Cheong

**Affiliations:** 1 School of Physical and Mathematical Sciences, Nanyang Technological University, 21 Nanyang Link, Singapore 637371, Singapore; 2 Complexity Institute, Nanyang Technological University, 61 Nanyang Drive, Singapore 637335, Singapore; 3 Department of Computational Intelligence, Faculty of Computer Science and Management, Wrocław University of Science and Technology, Ignacego Łukasiewicza 5, 50-371 Wrocław, Poland

**Keywords:** SciSci, knowledge evolution, machine learning

## Abstract

The advancement of science, as outlined by Popper and Kuhn, is largely qualitative, but with bibliometric data, it is possible and desirable to develop a quantitative picture of scientific progress. Furthermore, it is also important to allocate finite resources to research topics that have the growth potential to accelerate the process from scientific breakthroughs to technological innovations. In this paper, we address this problem of quantitative knowledge evolution by analyzing the APS data sets from 1981 to 2010. We build the bibliographic coupling and co-citation networks, use the Louvain method to detect topical clusters (TCs) in each year, measure the similarity of TCs in consecutive years, and visualize the results as alluvial diagrams. Having the predictive features describing a given TC and its known evolution in the next year, we can train a machine learning model to predict future changes of TCs, i.e., their continuing, dissolving, merging, and splitting. We found the number of papers from certain journals, the degree, closeness, and betweenness to be the most predictive features. Additionally, betweenness increased significantly for merging events and decreased significantly for splitting events. Our results represent the first step from a descriptive understanding of the science of science (SciSci), towards one that is ultimately prescriptive.

## 1. Introduction

We all become scientists because we want to create an impact and make a difference to the lives of those around us and also to the many generations that are to come. We all strive to make choices in the problems we study, but not all choices lead to breakthroughs. There is actually much more about scientific breakthroughs that we can try to understand. For one, science is an ecosystem of scholars, ideas, and papers published. In this ecosystem, scientists can form strongly interacting groups over a particular period to solve specific problems, but later drift apart as their interests diverge, or due to the availability or paucity of funds, or other factors. The evolution of these problem driven groups is more or less completely documented by the papers published as outcomes of their research. By analyzing groups of closely related papers, researchers could extract rich information about knowledge processes [1,2,3,4]. The potential to map scientific progress using publication data has attracted enormous interest recently [5,6,7]. However, compared to the study of science at the level of individual papers [8,9,10] and at the level of the whole citation network [11,12,13,14,15], where much work has already been done, the research on science at the community level is still limited [1,3,16,17].

In a recent paper, Liu et al. demonstrated the utility of visualizing and analyzing scientific knowledge evolution for physics at the aggregated mesoscale through the use of alluvial diagrams [3]. In this picture, papers are clustered into groups (or communities), and these groups can grow or shrink, merge or split, new groups may arise, while the others may dissolve. This shares a very strong parallel with what some researchers discovered in social group dynamics [18]. More importantly, many breakthroughs were made by scientists absorbing knowledge from other fields, often in a very short time. On the alluvial diagrams, these knowledge transformations manifest themselves as merging and splitting events. Clearly, funding agencies, universities, and research institutes would want to promote growing research fields, and particularly those where breakthroughs are imminent. This is why it is important to be able to predict future events. Liu et al. [3] attempted this in their paper by analyzing the correlation between event types and several network metrics. Unfortunately, such predictions are very noisy. While merging events are highly correlated with interconnections between communities, the correlation between splitting events and the internal structure of communities are much more complex; besides, the predictions of forming, dissolving, growing, and shrinking were not considered at all.

Given the recent successes in the area of machine learning and artificial intelligence in a variety of prediction problems [19,20], as well as having developed and validated a general framework to predict social group evolution in Saganowski et al. [21], we decided to utilize machine learning techniques to fill the gap in predicting scientific knowledge events [22,23,24]. The overall idea behind the group evolution prediction (GEP) method is to build a classification model trained with historical observations in order to predict the future group changes based on their current characteristics, such as size, density, the average degree of nodes, etc. A single historical observation consists of a set of features describing the group at a given point in time and an event type that this group just experienced. The profile of the group may reflect its structure (e.g., density), dynamics (e.g., average age of its member articles), or context (e.g., the journals from which the articles (group members) come). In total, we used over 100 features, some of which were already known to the literature, whereas the others focusing on the dynamics and context were the new, unique features proposed in this paper. Indeed, when we ranked the most valuable features contributing to the successful prediction of knowledge evolution events, the new features were among the best ones. In order to be able to perform the prediction of future group changes, we have to track and learn the model on the historical cases. For that purpose, the group changes from the past (historical evolution) need to be defined and discovered using the methods successfully applied in the social network analysis field, e.g., the GED method [25], Tajeuna et al.’s method [26], or others [27]. Most of the methods consider the similarity between the groups in the consecutive time windows as a major factor to match similar groups and further to identify the evolution event type between them. In our work, we apply the GED method, which facilitates both the group quantity (the number of common members) and the group quality (the importance of common members), in order to match related groups. This allows us to enrich the co-citation evolution network with information about member relations, which is depicted in the social position measure [28].

In this study, we extract groups (topical clusters (TCs)) from the bibliographic coupling networks (BCNs) and independently from the co-citation networks (CNs) for the period 1981–2010. Next, the GED method is utilized to label four types of evolution events (changes of TCs): continuing, dissolving, merging, and splitting. Then, we use an auto-adaptive mechanism to find the most predictive machine learning model, together with its parameters for each network. Additionally, two scenarios were considered for each network: when the number of events of each kind is imbalanced (the original case) and balanced by equal sampling. In general, the prediction quality was satisfactory and good for all event types, with F-measures substantially exceeding 0.5. Such values are significantly greater than the baseline F-measures of 0.14–0.21 for both networks. The feature ranking tells us that the most informative features are context based like the number of PRE, PRB, and RMP papers belonging to the group and the structural features like the degree, closeness, and betweenness. While looking more carefully at the betweenness of papers from two merging TCs, we found significantly higher betweenness for papers that are linked across these two TCs than those connected inside the TCs. No such enhancement in betweenness was found for continuing TCs, while a significant decrease in average betweenness was found for splitting TCs. In summary, our findings suggest that evolutionary events in the landscape of physics research can be predicted accurately using various machine learning models, and understanding this predictive power in terms of important features is a worthwhile future research direction.

## 2. Materials and Methods

The entire analytical process consists of several steps that are primarily defined by the group evolution prediction (GEP) framework. First, the bibliographic coupling network (BCN) and co-citation network (CN) are extracted from the references placed in the papers from a given time window (see Figure 2), and this is carried out separately for each period.

As a result, we get a time series of BCNs/CNs. Next, paper groups called topical clusters (TCs) are extracted using the Louvain clustering methods, independently for each BCN/CN in the time series. Each group is described by the set of predictive features. Having TCs for consecutive periods, we were able to identify changes in TC evolution using the group evolution discovery (GED) method that appropriately labels the TC changes; see below.

**Figure 1:**
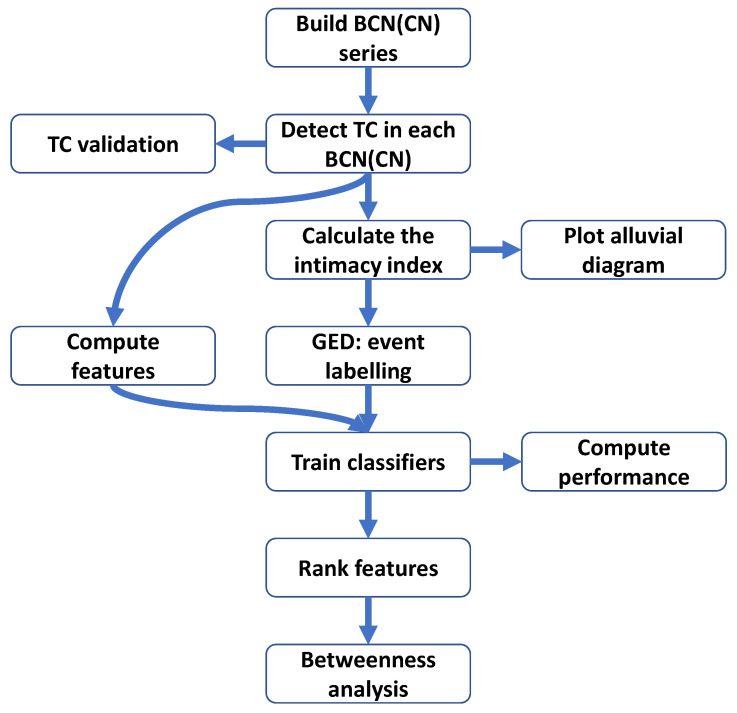
The workflow of this paper. TC, topical cluster.

Independently, the features’ ranking and its validation were performed to find the most valuable TC measures. Based on this ranking, a structural measure node betweenness was selected for the more in-depth studies as the early signal for splitting or merging. The above-mentioned steps are summarized and visualized in Figure 1.

### 2.1. GEP Method

The group evolution prediction (GEP) method is the first generic approach for the prediction of the evolution of groups [21]; in our case, groups correspond to TCs. The GEP process consists of six main steps: (1) time window definition, (2) temporal network creation, (3) group detection, (4) group evolution tracking, (5) evolution chain identification and feature calculation, and (6) classification using machine learning techniques. Thanks to its adaptable character, we were able to apply it to the BCN and CN differently. For the group (TC) detection in both networks, we applied the Louvain method [29]. The group evolution tracking was performed with the GED method (see below), but we used different similarity measures for each network BCN and CN (see below). The set of features describing the group at a given time window was adjusted to our networks, as some of the features defined in the GEP method were not applicable in our case. We also introduced some new, dedicated measures appropriate for bibliographical data; see SI (Supplementary Information) for the complete list. Finally, we applied the Auto-WEKA tool to find the best predictive model and its parameters from a wide range of all possible solutions. The commonly known average F-measure was used as a prediction performance measure. The stratified sampling and 10-fold cross-validation techniques were used to validate the model. The feature selection technique was applied to prevent model overfitting.

### 2.2. Bibliographic Coupling Network and Co-Citation Network

**Figure 2:**
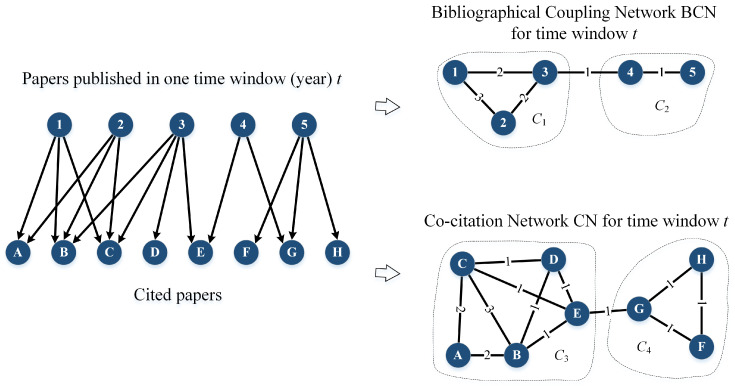
The process of building a bibliographical coupling network (BCN) and co-citation network (CN) from the citation bipartite network for a given period: year *t*. Both BCN and CN are undirected and weighted; the weights denote the number of shared citations (BCN) or co-citing papers (CN). Separate topical clusters are extracted for BCN ($C_{1}$, $C_{2}$) and CN ($C_{3}$, $C_{4}$). Nodes with numbers are papers from a given period being considered, and nodes with letters are their references.

In the BCN and CN, nodes represent papers, and undirected but weighted edges denote the bibliographic coupling strengths and co-citation strengths, respectively. That is, if two papers share *w* common references, the BCN edge between them would have a weight of *w*. For example, Papers 1 and 2 in Figure 2 share three citations: A, B, and C, whereas Papers 3 and 4 commonly cite only one paper: E. On the other hand, if two papers are cited together by $w^{\prime }$ papers, the edge between them in the CN receives weight $w^{\prime }$. Papers A and B are cited together by two other papers: 1 and 2, but Papers B and C by three, i.e., additionally by Paper 3. Both BCN and CN are temporal networks, in which the nodes are all papers published (BCN) or papers cited (CN) within a specific time window. We assumed that the reasonable time window for bibliographical data was one year to facilitate the analysis of changes in scientific knowledge, i.e., changes in topical clusters year-by-year. For the BCN, only the giant component, which in most cases occupied 99% of the whole BCN, would be considered for the TC detection and evolution analysis. For the CN, we did not use all papers cited in the given time window because most of them were cited only a small number of times, and thus, they had little influence on the broader knowledge evolution. Therefore, we ranked all available *N* papers $p_{1},p_{2},\ldots ,p_{N}$ in descending order by the number of times they were cited in this time window (year): $f_{1},f_{2},\ldots ,f_{N},f_{1}\geq f_{2}\geq \ldots \geq f_{N}$. Next, we chose the top *n* papers $p_{1},p_{2},\ldots ,p_{n}$ that totally gathered $\frac{1}{4}$ of all citations, i.e., such that n<N was the smallest integer to satisfy $\sum_{i=1}^{n}f_{i}\geq \frac{1}{4}\sum_{j=1}^{N}f_{j}$. The data we used in this paper were the APSdataset, consisting of about half a million publications between 1893 and 2013 and six million citation relations among them [30].

### 2.3. Community Detection and Validation

There are many approaches to community detection, including modularity based algorithms, hierarchical clustering, non-negative matrix factorization, principal component analysis, link partitioning, and others [31]. In this work, we used the Louvain method [29] to extract community structure from BCNs and CN. The community partitions we obtained in BCNs and CNs had considerably high modularities (about 0.75), which suggest clear and robust community structures. Furthermore, a different community detection algorithm was also used, i.e., Infomap, which gave very similar results as the Louvain method. For instance, the normalized mutual information between community partitions of BCN in 1991 from the Louvain method and Infomap algorithm was 0.66, which confirmed the existence and robustness of community structure in BCNs and CNs. In this study, we only used the Louvain method; however, the results were similar if we switched to Infomap or other community detection algorithms.

**Figure 3:**
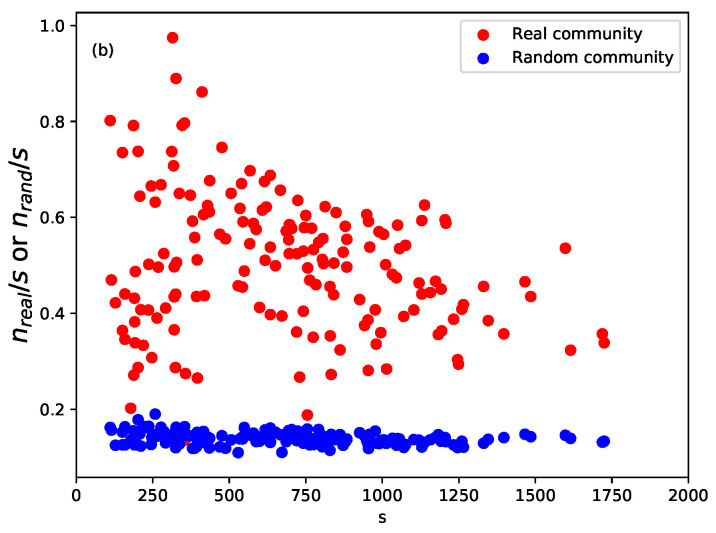
Comparison of PACS homogeneity between real BCN TCs, which are between 1991 and 2000 and have more than 100 papers, and their corresponding random collections. The fraction of the largest subset of papers sharing at least one PACS number as a function of *s* for real communities in the BCN and random collections. For clarity, the error bars are not shown in the figures because they are smaller than the marker size.

To verify that the communities extracted were really focused on closely related questions, we checked the Physics and Astronomy Classification Scheme (PACS) numbers of members of the communities. This cross-validation was independent of network structure; therefore, it provided more evidence for the robustness of TCs. In our study, we only used the first two digits of the PACS numbers, as a balance between accuracy and coverage. To test whether the PACS numbers appearing in the communities could have occurred due to randomness, we chose one year *t*, built its BCN, extracting the community structure with sizes $\left\{s_{1},s_{2},\ldots ,s_{n}\right\}$, and then randomly assigned papers in year *t* into *n* pseudo-communities of the same sizes, to remove any potential size effects. The results showed that the papers in the same community significantly focused on a small number of PACS numbers compared with a null model; see Figure 3. Interested readers can get more details on the systematic validation of TC in Liu et al. [3].

### 2.4. Intimacy Indices

To analyze the evolution of TCs, we needed to match them from consecutive years. The set of cited papers to a large extent overlapped year-by-year, so for the CN, we could use the regular approach proposed together with the GED method; see below and Brodka et al. [25]. For BCN, however, there was no overlap at all between papers published in the successive years because every paper could be published only once and in only one year. Even if we did not have the corresponding papers in TCs from two BCNs, i.e., two years, the papers’ references overlapped each another. Therefore, we could measure the similarity of their reference pools to reflect their inheritance. For that purpose, we introduced the forward intimacy index and backward intimacy index in Liu et al. [3]. The idea behind intimacy indices is that the references related to a particular topic change gradually. The forward intimacy index $I_{mn}^{f}$ and the backward intimacy index $I_{mn}^{b}$ between TCs $C_{m}^{t}$ in year *t* and $C_{n}^{t+1}$ in year t+1 are defined as follows:

(1) $$\begin{array}{cc} I_{mn}^{f} & =\sum_{i}\frac{N\left(R_{i},R_{n}^{t+1}\right)}{N\left(R_{i},R^{t+1}\right)}\frac{N\left(R_{i},R_{m}^{t}\right)}{L\left(R_{m}^{t}\right)},\\ I_{mn}^{b} & =\sum_{i}\frac{N\left(R_{i},R_{m}^{t}\right)}{N\left(R_{i},R^{t}\right)}\frac{N\left(R_{i},R_{n}^{t+1}\right)}{L\left(R_{n}^{t+1}\right)}. \end{array}$$

Here, the TCs at *t* and t+1 are $C^{t}=\left\{C_{1}^{t},\ldots ,C_{m}^{t},\ldots ,C_{u}^{t}\right\}$ and $C^{t+1}=\left\{C_{1}^{t+1},\ldots ,C_{n}^{t+1},\ldots ,C_{v}^{t+1}\right\}$, and we denote the references cited by papers in $C_{m}^{t}$ and $C_{n}^{t+1}$ as $R_{m}^{t}=R\left(C_{m}^{t}\right)=\left[R_{m1},\ldots ,R_{mp}\right]$ and $R_{n}^{t+1}=R\left(C_{n}^{t+1}\right)=\left[R_{n1},\ldots ,R_{nq}\right]$; $R^{t}=\left\{R_{1}^{t},\ldots ,R_{m}^{t},\ldots \right\}$. N(element,list) is the number of times element occurs in list, and L(list) is the length of list. For more details and examples of intimacy indices, please refer to Liu et al. [3].

### 2.5. GED Method

The group evolution discovery (GED) method [25] was used for tracking group evolution for historical cases to learn the classifier and for testing cases to validate classification results. The GED method makes use of the similarity between groups in the following years, as well as their sizes to label one of six event types: continuing, dissolving, merging, splitting, growing, and shrinking. However, we have adapted the GED method to label only four types of events: continuing, dissolving, merging, and splitting, as these were the most important to us. The other two (growing and shrinking) were covered by continuing. In general, the GED method allowed us to use various metrics as a similarity measure between groups. Therefore, the intimacy indices defined in Equation (1) were used for the BCN to match similar groups in the consecutive time windows. However, the original GED inclusion measures were used for the CN. This means that the similarity between two groups from two successive time windows was reflected by the inclusion measure, which was calculated for two scenarios: inclusion $I(C_{n}^{t},C_{m}^{t+1})$ of a group $C_{n}^{t}$ from time window *t* in another group $C_{m}^{t+1}$ from time window t+1 (forward; Equation (2)) and inclusion $I(C_{m}^{t+1},C_{n}^{t})$ of this second group $C_{m}^{t+1}$ from t+1 in the first group $C_{n}^{t}$ from *t* (backward; Equation (3)). The inclusion measure makes use of the social position SP(p), which is a kind of weighted PageRank. It denotes the importance of paper *p* being cited among all other papers [28]. The inclusions for CN are defined as follows:

(2) $$I\left(C_{n}^{t},C_{m}^{t+1}\right)=\overset{\mathrm{group}\;\mathrm{quantity}}{\overset{︷}{\frac{∥C_{n}^{t}\cap C_{m}^{t+1}∥}{∥C_{n}^{t}∥}}}\cdot \underset{\mathrm{group}\;\mathrm{quality}}{\underset{︸}{\frac{\sum_{p\in (C_{n}^{t}\cap C_{m}^{t+1})}SP\left(p\right)}{\sum_{p\in (C_{n}^{t})}SP\left(p\right)}}}\cdot 100\%,$$

(3) $$I\left(C_{m}^{t+1},C_{n}^{t}\right)=\overset{\mathrm{group}\;\mathrm{quantity}}{\overset{︷}{\frac{∥C_{m}^{t+1}\cap C_{n}^{t}∥}{∥C_{m}^{t+1}∥}}}\cdot \underset{\mathrm{group}\;\mathrm{quality}}{\underset{︸}{\frac{\sum_{p\in (C_{m}^{t+1}\cap C_{n}^{t})}SP\left(p\right)}{\sum_{p\in (C_{m}^{t+1})}SP\left(p\right)}}}\cdot 100\%.$$

If both inclusions (CN) or both intimacy indices (BCN) are greater than the percentage thresholds alpha and beta (the only parameters in this method), the method labels the event continuing. If at least one inclusion or one intimacy index exceeds one of the thresholds, the splitting and merging events considered, the proper event is assigned depending on the number of similar groups in *t* and t+1. If both inclusions or both intimacy indexes are below the thresholds, i.e., the group has no corresponding group in the next time window, the dissolving event is assigned.

### 2.6. Feature Ranking

Rankings of the most prominent features were obtained by repeating the feature selection 1000 times using a basic evolutionary algorithm [32], as proposed in Saganowski et al. [21]. The alternative approach would be to use the forward or backward feature elimination technique, but our own implementation gave us more flexibility and control over the experiment. The rankings were received for the 30 year span (1981–2010). Next, only the top 10 features were selected to described TCs in two additional years (2010–2012) and predict TC evolution. The results revealed the superiority of feature selection compared to the raw approach with all features’ engagement.

## 3. Results

### 3.1. Physics Research Evolution for 1981–2010

We begin with studying how scientific knowledge evolved in terms of communities of research papers and how these communities changed over time. There were several studies on the evolution of knowledge within the set of whole journals [2], which was considered as the analysis on the macroscopic level. Furthermore, some research was carried out for the collection of papers, usually involving some subjective criterion provided by the authors, e.g., only papers cited at least 100 times [1]. As a result, they focused only on a small subset: the most prominent, frequently cited papers, which do not represent the whole diverse domain knowledge. This kind of analysis was considered as microscopic. In our approach, we assumed that the most informative way was to analyze neither the entire journal, nor the most cited papers, but whole communities of closely related papers. These communities emerged naturally since they shared the same citation patterns. The analysis at such a level provided a better balance between high and low granularity. We called this kind of analysis mesoscopic because it was in between the macroscopic scale of journals and the microscopic scale of individual papers. However, if we performed community detection directly on the citation network, we might end up with communities consisting of both old and recent papers simultaneously. In such a case, it is difficult to interpret how scientific knowledge has evolved from the past to the present. We should be able to explain that such and such communities represent scientific knowledge from an earlier year, whereas the other communities correspond to scientific knowledge from another consecutive year. This enabled us to compare them and to distil a picture of how scientific knowledge has evolved from past to present. It required, however, constructing the networks from research papers that were published in a given year (bibliographic coupling) or papers that were cited in a given year (co-citation). The bibliographic coupling network (BCN) reflected the relation between present publications, while the co-citation network (CN) represented the relation between papers that had a strong influence on recent publications. In this way, we could detect communities over the years and study how they evolved year-by-year; see the Methods Section for details on BCN and CN.

After building BCN and CN, the Louvain method was used to extract the community structures. By checking the Physics and Astronomy Classification Scheme (PACS) numbers of the papers in these communities, we showed that the BCN communities were meaningful and reflected the real structure of the scientific communities. These results suggested that the papers in the same community were very similar to each other in terms of research topic. These results suggest papers in the same community had high similarity to each other in terms of research topic. The method and results of the validation are briefly reviewed in the Methods Section; the interested reader is referred to Liu et al. for details [3]. For the CN communities, this validation is tricky because of two problems: (i) the old physics review papers had no PACS numbers, and (ii) PACS was revised several times, so the same numbers in different versions can potentially refer to different topics, or the same topics are referred to by different numbers in different versions. Nevertheless, systematic validation seemed to be impossible, although a quick check on some CN communities after 2010 suggested that the CN community structure also reliably reflected the actual scientific community. We refer to these validated units of knowledge evolution as topical clusters (TCs) in this paper.

**Figure 4:**
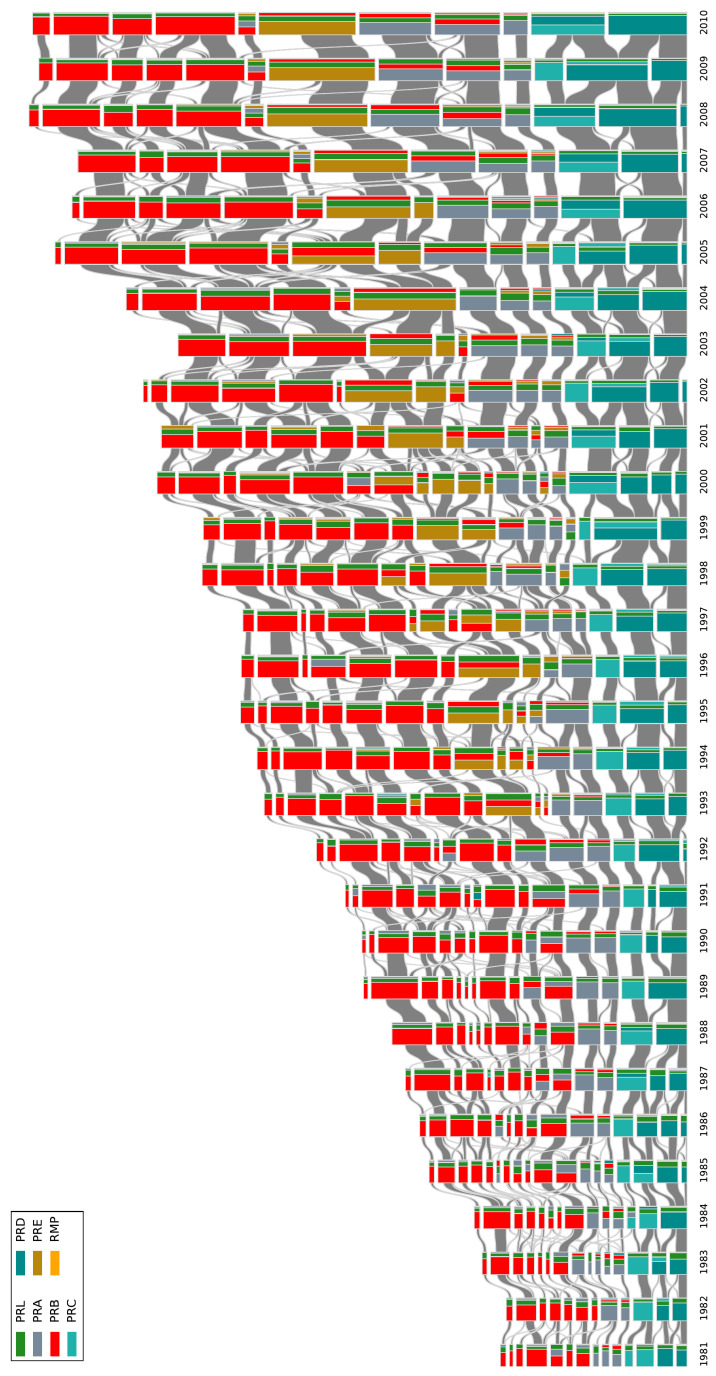
The alluvial diagram of APSpapers from 1981 to 2010 for the BCNs. Each block in a column represents a TC, and the height of the block is proportional to the number of papers in the TC. For clarity reason, only TCs comprising more than 100 papers are shown. TCs in successive years are connected by streams whose widths at the left and right ends are proportional to the forward and backward intimacy indices. The colors inside a TC represent the relative contributions from different journals.

In Figure 4, we provide the alluvial diagram that depicts the evolution of TCs within the BCNs for the period between 1981 and 2010. The equivalent alluvial diagram for the CNs is shown in Figure S2 in the SI. In both alluvial diagrams, we visualize the sequences of TCs, their inheritance relations, which can be intimacy indices (for the BCN communities), a fraction of common members or inclusion measures (for the CN communities), and the evolution processes they undergo; see the Methods Section for more details. The events (changes) that we can discern from the alluvial diagram (shown in Figure 4) are analogous to those recognized in social group evolution [18]. They represent forming, dissolving, growing, shrinking, merging, and splitting. We found in Liu et al. that the prediction of such events was hard since the correlation between them was nonlinear and complex. This challenge is addressed in the following section by tapping into the power of machine learning.

### 3.2. Event Labeling

The GED method takes into account the size and the similarity between groups (TCs) in the consecutive time frames in order to label groups’ changes (assign event type). There are four events considered in this work:

- Continuing: A research field is said to be continuing when the problems identified and solutions obtained from one year to another are of an incremental nature. It is likely to correspond to the repeated hypothesis testing picture of the progress of science proposed by Karl Popper [33]. Therefore, in the CN, this would appear as a group of papers that are repeatedly cited together year-by-year. In the BCN, this shows up as groups of articles from successive years sharing more or less the same reference list.
- Dissolving: A research field is thought to disappear in the following year if the problems are solved or abandoned, and no new significant work is done after this. For the CN, we will find a group of papers that are cited up to a given year, but receiving very few new citations afterwards. In the BCN, no new relevant papers are published in the field; hence, the reference chain terminates.
- Splitting: A research field splits in the following year, when the community of scientists who used to work on the same problems starts to form two or more sub-communities, which are more and more distant from one another. In terms of the CN, we will find a group of papers that are almost always cited together up till a given year, breaking up into smaller and disjoint groups of papers that are cited together in the next year. In the BCN, we will find the transition between new papers citing a group of older papers to new papers citing only a part of this reference group.
- Merging: Multiple research fields are considered to have merged in the following year when the previously disjoint communities of scientists found a mutual interest in each other’s field so that they solve the problems in their own domain using methods from another domain. In the CN, we find previously distinct groups of papers that are cited together by papers published after a given year. In the BCN, newly published papers will form a group commonly citing several previously disjoint groups of older papers.

**Figure 5:**
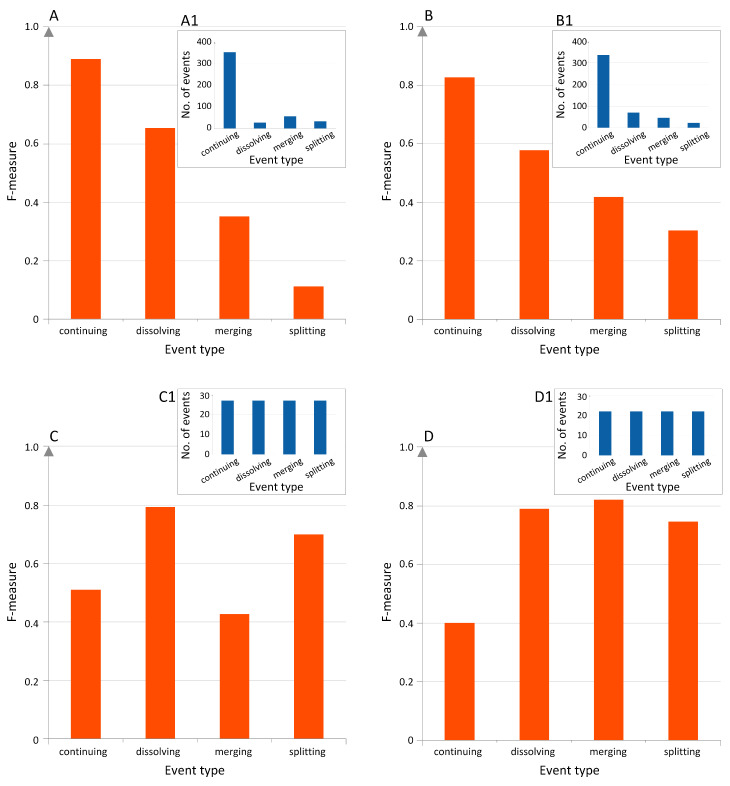
The prediction quality of classification results. The F-measure values for the imbalanced BCN (**A**) and CN (**B**) datasets, as well as the balanced BCN (**C**) and CN (**D**) datasets. The distribution of classes in the training sets is provided for each dataset: **A1**, **B1**, **C1**, **D1**, respectively. For the imbalanced datasets, the classifier focused on the dominating continuing event. Balancing the datasets increased the overall prediction quality by over 20%.

The GED method has two main parameters (alpha and beta), which are the levels of inclusion that groups in the consecutive years have to cross in order to be considered as matching groups. We applied the GED method with a wide range of these parameters from 5 to 100%. The characteristics of the considered networks required us to set the alpha and beta thresholds to very low values, i.e., 30% for the BCN and 10% for the CN; see SI for more details. In total, we obtained 479 various events for the BCN and 492 events for the CN, which were our observations and the labels in the prediction part of our study. In both networks, the distribution of the events was imbalanced with the continuing event dominating over all other types; see Figure 5(A1,B1).

### 3.3. Future Events’ Prediction

The machine learning approach to prediction requires dividing the data into two parts: the training dataset and test dataset. The training data are used to train the classifier, which can then label events in the test data. The labeled values are compared with the event labels, and the prediction performance is calculated. More than 450 observations were used to train the classifiers. Each observation contained 77 normalized features (preselected from the initial 100) divided into three categories: microscopic features (related to nodes in the group, e.g., node degree), mesoscopic features (related to the entire group, e.g., the group size), and macroscopic features (related to the whole network, e.g., network density). Mesoscopic features calculated for individual nodes are commonly aggregated for all nodes from the group, e.g., average node degree or betweenness in the group. See SI for the complete list of features used.To select the best classification algorithm (model) automatically, as well as its hyper-parameter settings to maximize the prediction performance, the Auto-WEKA software package [34] was utilized. For each network, we ran the Auto-WEKA for 48 h, which allowed us to validate nearly 20,000 configurations per network. The metric being maximized was the F-measure, commonly used for multi-class classification. The overall classification quality was calculated as the average F-measure for all event types, treating them as equally important.

The predicted output variable (event labels) had an imbalanced distribution. Commonly, classifiers tend to focus on the dominant event type (class), which is very well predicted, but at the expense of the minority event types. For the imbalanced BCN dataset, the best performance was achieved with the attribute selected classifier (with the SMOas a base classifier), which performed feature selection [35]. The percentage of the correctly classified instances was 80.6%, while the average F-measure was only 0.50 due to classifier focusing on continuing, which was the most frequently occurring event type; see Figure 5A. For this event, the F-measure value was equal to 0.89, and only seven events out of 352 were incorrectly classified. The worst classified was the splitting event, whose F-measure was only 0.11. Most of the splitting events were incorrectly classified as continuing (31 out of 33 events). The second worst was merging, with the F-measure of 0.35. Again, the majority of the merging events were wrongly classified as continuing events: 38 out of 56. Interestingly, the splitting and merging events were never cross-classified mistakenly. For the imbalanced CN dataset, the best performance was achieved with a lazy classifier, which used locally weighted learning [36]. The percentage of the correctly classified instances was 73.3%, while the average F-measure was only 0.53, again due to the classifier concentrating on the dominating continuing event type; see Figure 5B. The F-measure value for the continuing event was only 0.83; however, as many as 50 continuing events (out of 337) were wrongly classified as dissolving. Similar to BCN, many splitting and merging events were incorrectly classified as continuing: 17 out of 22 events and 24 out of 46 events, with the F-measure equal to 0.30 and 0.42, respectively.

By balancing the imbalanced training datasets (i.e., by under-sampling them), we forced the classifiers to pay more attention to the features rather than to the number of occurrences of the particular majority event type. Please note that the test set was untouched, i.e., left imbalanced. As a result of balancing datasets, the previously minor event types (dissolving, merging, and splitting) were predicted much better, but with a significant drop in performance of the continuing event classification. More importantly, by balancing the datasets, we increased the overall prediction quality by over 20%. For the balanced BCN dataset, the best performance was achieved by means of the boosting based classifier AdaBoost with Bayes net as the base model. The percentage of the correctly classified instances was 62.0%, and the average F-measure was 0.61. The biggest sources of errors were merging events, which were wrongly classified as continuing and dissolving, as well as continuing wrongly classified as splitting. The best classified event was dissolving (only four mistakes in 27 classifications; the overall score was 0.79), followed by the splitting event (six mistakes in 27 classifications; overall F-measure of 0.70). For the balanced CN dataset, the attribute selected classifier (with the PART [37] as a base classifier) provided the best results: the percentage of the correctly classified instances was 69.32%, while the average F-measure was 0.69. The dissolving, merging, and splitting events were classified very well with the F-measure values equal to 0.79, 0.82, and 0.75, respectively. Most of the continuing events were wrongly classified as splitting (13 out of 22), which resulted in a lower F-measure value of 0.40.

What is interesting for us to note is that the prediction results for the CN were slightly better than for the BCN. A possible explanation is that for the CN, we used a richer similarity measure containing users’ importance information. Thus, the event tracking and, therefore, the ground truth could be more accurate. Overall, the prediction quality expressed by the average F-measure was very good for the imbalanced, as well as for the balanced datasets, as the baseline results obtained with the ZeroR classifier were much worse: F-measure of 0.21 for both BCN and CN imbalanced datasets, 0.18 for the balanced BCN, and 0.14 for the balanced CN. For each dataset, different classifiers turned out to be the best; however, most models were wrapped with the boosting or meta classifiers.

### 3.4. Predictive Feature Ranking

**Figure 6:**
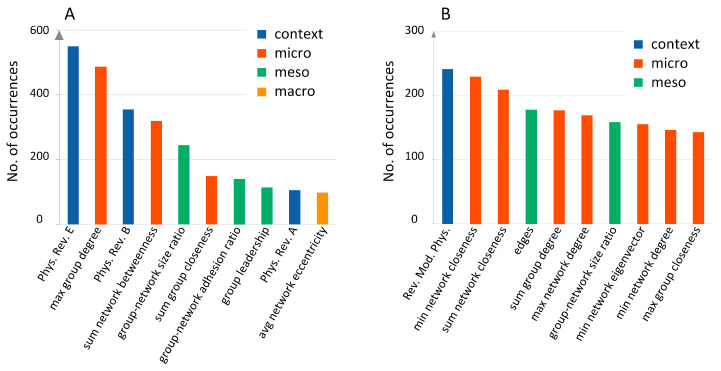
Feature ranking. The most frequently selected features in 1000 iterations for the BCN (**A**) and CN (**B**) datasets. The context based features (number of papers published in a given journal) turned out to be the most informative, followed by the microscopic structural measures, especially closeness, degree, and betweenness.

The feature selection technique is used in machine learning to find the most informative features, to avoid classifier overfitting, to eliminate (or at least to reduce) the noise in the data, as well as to provide some explanations about phenomena [32]. By repeating the feature selection 1000 times, we obtained 1000 sets of selected features. Next, we calculated how many times each feature was selected, thus receiving the ranking of the most often selected features. For the BCN, the context-based features dominated the ranking. It referred especially to the number of papers from the Physical Review E, Physics Review B, and Physical Review A; see Figure 6A. Besides the context, the network features based on degree, betweenness, size, and closeness measures were most informative, which tells us that the structural properties were as important as context awareness. The context based feature, i.e., the number of papers published in the Review of Modern Physics, was the most often selected for the CN dataset. It was followed by closeness and degree based features in the ranking; see Figure 6B. For both networks, macroscopic features were ranked rather low, which suggests that the general network profile was not very important, perhaps because of the smooth changes in the entire network. Surprisingly, the dynamic features, e.g., related to the average age of references (for BCN) and age of articles (for CN), did not show an informative value and were ranked very low for both networks. The rankings were validated in the additional two years of data available (2010–2012). The prediction was performed twice: (i) using all features and (ii) using the top 10 ranked features only. Selecting only the top 10 features boosted the quality of the prediction by 11% for the CN and by 2% for the BCN, which underlined the necessity of the feature selection process.

### 3.5. Changes to the Betweenness Distributions Associated with Merging and Splitting Events in BCN

**Figure 7:**
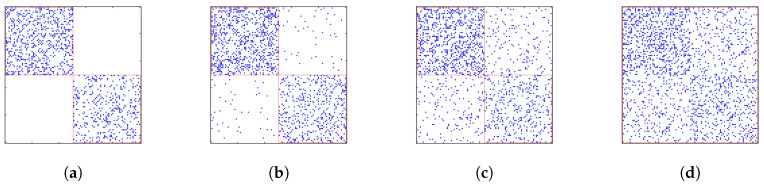
Part of the BCN adjacency matrix for two TCs (red boxes) that ultimately merged. (**a**) No links between the two TCs at first. (**b**) Few links between the two TCs. (**c**) More links between the two TCs. (**d**) Many links between the two TCs, leading to their identification as a single merged TC (big red box) by the Louvain method.

Having the list of best predictive features (Figure 6), we can analyze some of them more in-depth to look for early warning signals. Basically, we believe that scientific knowledge evolves slowly, and this slow evolution drives the evolution of citation patterns. Therefore, there must be specific changes in citation patterns that precede merging and splitting events. Besides the number of PRE papers in a TC, sum_network_betweennessis also a strongly predictive feature; see Figure 6A. This suggests that we should look at the betweenness of papers in the BCN more carefully. The betweenness of the node denotes what percentage of the shortest paths between all pairs of nodes in the network passes a given node. Values of nodes’ betweenness can be aggregated (sum, average, max, min) for all nodes in the TC, as we list in Table S1 in SI. However, in this section, we only focus on the distribution of the original node betweenness. Naively, when we considered the part of the BCN adjacency matrix corresponding to two TCs that ultimately merged, we expected to find few links between TCs at first. However, as the number of links between TCs increased over time, the modularity-maximizing Louvain method would eventually merge the two TCs into a single TC. This is shown schematically in Figure 7, where in general, betweenness would increase on average with time as the two TCs merge.

In reality, there are always links between TCs, and the numbers and strengths of these links fluctuate over time. To develop a more quantitative description of the merging events outlined in Figure 4, as well as splitting and continuing events, we focused on five events going from 1999 to 2000, shown in Table 1.

#### 3.5.1. **1999.01** + **1999.02** → **2000.03**

**Figure 8:**
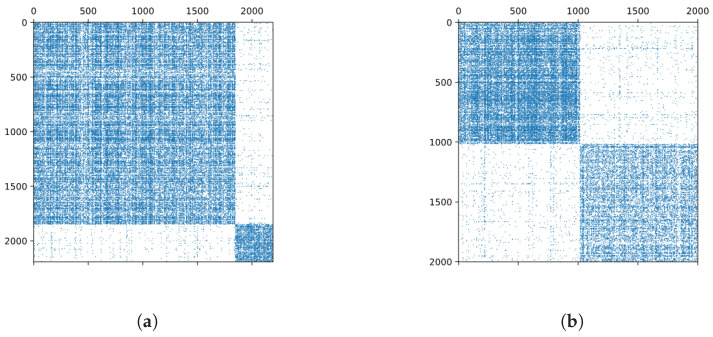
(**a**) The adjacency matrix of the BCN associated with the TCs 1999.01 (top dense block) and 1999.02 (bottom dense block). (**b**) The adjacency matrix of the BCN associated with the TCs 1999.11 (top dense block) and 1999.12 (bottom dense block).

Let us consider the part of the BCN associated with the TCs. For example, for 1999.01 and 1999.02, we can see from Figure 8a that connections within 1999.01 and 1999.02 were very dense, but there were also some links between the two TCs. In fact, we found 164 out of 1849 papers in 1999.01 with non-zero bibliographic coupling to 144 papers in 1999.02 (344 papers).

The natural question we then ask is: are the betweennesses of the 164 papers in 1999.01 that are coupled to 1999.02 larger, equal to, or smaller than the betweenness of the rest of the 1685 papers in 1999.01 not coupled to 1999.02? Alternatively, if we think of the 164 papers as randomly sampled from the 1849 papers in 1999.01, are we sampling the 164 betweenness in an unbiased fashion? To distinguish the different parts of the TC, we call all papers in 1999.01 that have coupling with papers in 1999.02 as 1999.01*a* and the rest of the papers as 1999.01*b*. For more detail analysis, we will divide 1999.01*a* and 1999.01*b* into 1999.01aα, 1999.01aβ, 1999.01bα, and 1999.01bβ. 1999.01aα consists of 17 papers in 1999.01a that do not have references in common with papers in 1999.01b; 1999.01aβ consists of 147 papers in 1999.01a that have references in common with papers in 1999.01b; 1999.01bα are 907 papers in 1999.01b that have references in common with papers in 1999.01a; and 1999.01bβ represents 778 papers in 1999.01b that do not have references in common with papers in 1999.01a.

**Figure 9:**
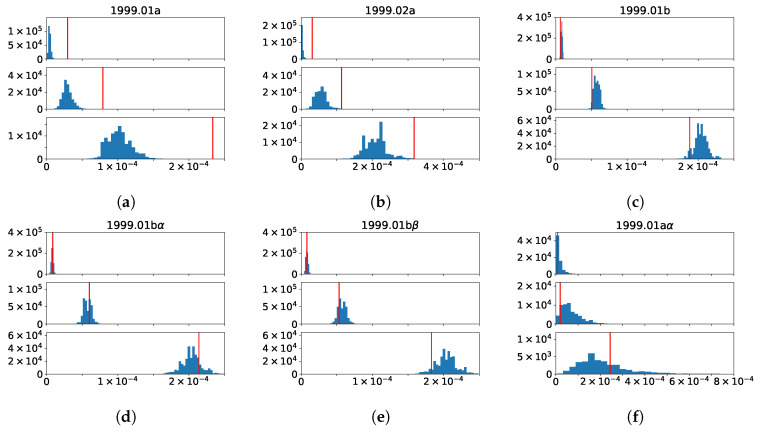

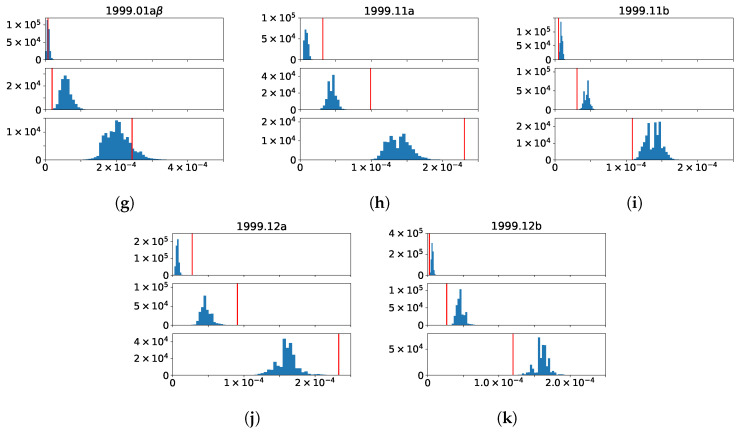
The lower (top), median (middle), and top quartile (bottom) of the betweennesses in (**a**) 1999.01a, (**b**) 1999.02a, (**c**) 1999.01b, (**d**) 1999.01bα, (**e**) 1999.01bβ, (**f**) 1999.01aα, (**g**) 1999.01aβ, (**h**) 1999.11a, (**i**) 1999.11b, (**j**) 1999.12a, and (**k**) 1999.12b shown as red vertical lines and 10^6^ random samples of the same number of betweennesses from 1999.01 (**a**,**c**–**g**), or 1999.02 (**b**), or 1999.11 (**h**,**i**), or 1999.12 (**j**,**k**) shown as blue histograms.The x-axes are “quartile value”, and y-axes are “null model density”.

In Table 2, we show the 25th, 50th, and 75th percentiles of the papers in these smaller groups, compared to those of the 1849 papers in 1999.01 and the 344 papers in 1999.02. As we can see, the 25th, 50th, and 75th percentile betweenness in the connecting parts (1999.01a and 1999.02a) were all higher than the 25th, 50th, and 75th percentile betweenness in the non-connecting parts (1999.01b and 1999.02b). More importantly, these percentile betweenness were higher than the 25th, 50th, and 75th percentile betweenness of the TCs 1999.01 and 1999.02 themselves. To test how significant these quartiles were in 1999.01a, we randomly sampled 164 betweenness values from 1999.01 10^6^ times and measured the quartiles of these samples. When we draw random samples from a TC, the 25th, 50th, and 75th percentiles depend on the size of the TC. There as more variability in these quartiles in smaller samples than in larger samples. Therefore, in the test for statistical significance, the observed quartile had to be tested against different null model quartiles for samples of different sizes. To do this, we drew samples with a range of sizes from the same set of betweenness and, for a given quartile (25%, 50%, or 75%), fit the minimum quartile value against the sample size to a cubic spline and the maximum quartile value against sample size to a different cubic spline. With these two cubic splines, we could then check whether the observed quartile value for a sample of size *n* was more than or less than the null model minimum or maximum using cubic spline interpolation. From the histograms shown in Figure 9a, we see that the betweenness quartiles of 1999.01a were statistically larger than random samples of the same size from 1999.01, at the level of *p* < 10^−6^, which means the papers in 1999.01a had significantly larger betweenness than other papers in 1999.01.

#### 3.5.2. **1999.01** → **2000.02** + **2000.03**

When a TC splits into two in the next year, we expect the links between two parts *a* and *b* in the TC to have thinned out to the point that the modularity *Q* of the whole is lower than the modularities $Q_{a}$ and $Q_{b}$ of the two parts. However, in general, we would not know how to separate the TC into the two parts *a* and *b*. Fortunately, for the 1999.01→2000.02+2000.03 splitting event, we also knew the part 1999.01a, which merged with 1999.02a, became 2000.03. Therefore, we might naively expect 1999.01b to be the part that split from 1999.01 to become 2000.02. If we test the quartiles of 1999.01b, against random samples of the same size from 1999.01, we find the histograms shown in Figure 9c. As we can see, the betweenness quartiles of 1999.01b were quite a bit lower than the typical values in 1999.01, but this difference was statistically not as significant as the quartiles of 1999.01a. Thinking about this problem more deeply, we realized that while papers in 1999.01b had no references in common with 1999.02, some of them did share common references with 1999.01a. Let us call these sets of papers 1999.01aα (papers do not have references in common with papers in 1999.01b), 1999.01aβ (papers have references in common with papers in 1999.01b), 1999.01bα (papers have references in common with papers in 1999.01a), and 1999.01bβ (papers that do not have references in common with papers in 1999.01a). In Figure 9d, we learn from the histograms that the betweenness quartiles of 1999.01bα are indistinguishable with random samples of the same size from 1999.01. On the other hand, from the histograms in Figure 9e, we find out that while the lower betweenness quartile of 1999.01bβ is indistinguishable with the random samples of the same size from 1999.01, its median and the upper quartile are both on the low sides of the random sample distributions. This suggests a split of 1999.01 to (1999.01a + 1999.01bα) + 1999.01bβ.

Just to be safe, we also checked the betweenness quartiles of 1999.01aα and 1999.01aβ, against random samples of the same sizes from 1999.01. As we can see from Figure 9f,g, the lower quartiles and medians are lower than those obtained from random samples, but the upper quartiles are decidedly higher. However, the difference between 1999.01aα and 1999.01aβ was not as obvious as the difference between 1999.01bα and 1999.01bβ, and one possible reason was the smaller sample size (17, 147 vs. 907, 778). Again, these results were consistent with the picture that the rise in betweenness in 1999.01a was driving the merging with 1999.02a, while the fall in betweenness in 1999.01bβ was driving a splitting inside 1999.01.

#### 3.5.3. **1999.11** + **1999.12** → **2000.15**

Although a small part split off from each of 1999.11 and 1999.12, the main event associated with the two TCs was a symmetric merging. Looking again into the relevant parts of the BCN, we found 299 out of 1014 papers in 1999.11 coupled to 347 out of 988 papers in 1999.12, and we called them 1999.11a and 1999.12a, respectively. As we can see from the histograms in Figure 9h,j, the betweenness quartiles in 1999.11a and 1999.12a were significantly higher than one would expect from random samples of 1999.11 and 1999.12. Simultaneously, the betweenness quartiles in 1999.11b and 1999.12b were significantly lower than in random samples of 1999.11 and 1999.12 (see Figure 9i,k). Therefore, what we see here might be the early warning signals of merging, as well as that of asymmetric splitting.

#### 3.5.4. **1999.04** → **2000.06** and **1999.13** → **2000.16**

So far, we have learned that a decrease in betweenness within a TC signals a possible split, whereas an increase in betweenness of the part of the TC coupled to another TC signals a merger between the two TCs. For this story to be consistent, we must not see these signals in the continuing events 1999.04→2000.06 and 1999.13→2000.16. However, if we go through the full BCN, we find that 370 out of 389 papers in 1999.04 and 308 out of 319 papers in 1999.13 are coupled to papers outside of these TCs, which suggests the possibility of merging or splitting.

However, as we can conclude from Table 3, while the lower betweenness quartiles of the coupling parts of 1999.04 and 1999.13 with other TCs may be significantly larger than those of random samples of the two TCs, the highest betweenness quartiles were never significantly larger. Therefore, at the same level of confidence that we have set for the precursors of merging between 1999.01 and 1999.02, as well as between 1999.11 and 1999.12, we have to say that there were no significant precursors for 1999.04 and 1999.13 to merge with other TCs.

What about splitting then? A TC is likely to split into two if at least one of two parts has reduced betweenness. We see in Table 3 that betweenness in the coupling parts of 1999.04 and 1999.13 was not significantly lower than that of random samples. Therefore, we looked at the non-coupling part, i.e., papers in 1999.04 and 1999.13, which had no references in common with papers in other TCs, but they may have common references with papers in the same TCs. We called these non-coupling parts 1999.04b and 1999.13b, respectively (the bottom row in Table 3). Only the top betweenness quartile of 1999.04b fell below that of random samples from 1999.04 in Table 3. Therefore, the early warning for a splitting event in the next year is not strong enough. For 1999.13b, on the other hand, all three betweenness quartiles fell below that of random samples from 1999.13, even after we accounted for the small size of 1999.13b. This suggests that the probability of a splitting event next year is high, but 1999.13 continued on to 2000.16, which thereafter continued to 2001 without merging or splitting. This might be because additional conditions, like the size of TC being large, must be satisfied before a splitting can occur.

## 4. Discussion and Conclusions

During the past two decades, researchers have made many efforts to understand the system of science. Many problems have been solved; however, the understanding of interactions between different fields is still limited. Investigating the temporal network (BCN, CN) and its community structures, we were able to measure and quantify the complex interaction between different fields, particularly in physics, over time. Naturally, we would like to have a predictive power based on this picture. However, the correlation between network structure and evolution events is nonlinear and complex. Therefore, we turned to machine learning techniques, which have shown a great power to solve predictive problems that are hard to solve using traditional statistical methods. To our knowledge, this is the first study that utilized both machine learning and network science approaches to predict the future of science at the community level.

To be able to identify changes in TCs, we needed to define time windows used for network creation and community detection. The natural choice for bibliographical data was the usage of single years, since the publishing process may last many months. Obviously, another detail may be considered like multiple years, e.g., two or five years. In our approach, i.e., both for BCN and CN, every citation had the same importance. However, there were some other concepts like fractional counting of citations [38]. It assumes that the impact of each citation is proportionate to the number of references in the citing document. Additionally, it can be differentiated depending on, e.g., the quality of the journal. For the CN, we calculated the similarity between groups in the consecutive time windows in two ways: (i) using the plain relative overlap measure and (ii) using the inclusion measure based on social position. The idea was to enrich evolution data with the structural information occurring between the nodes. It turned out that both measures provided similar labeling, but the evolution tracking with the social position information produced a slightly better initial prediction. Therefore, the study was continued only for the inclusion measure; see SI for more information.

We decided to analyze more in-depth only one feature describing the structural profile of TCs, namely node betweenness. It was primarily caused by the limited amount of resources and the complexity of the analyses. The entire process required much human assistance and could not have been easily automated. In our experiments, we utilized the raw, imbalanced or artificially flattened, balanced datasets. However, depending on the study purpose, we could bias some classes we were more interested in, e.g., split. This could be achieved either by means of appropriate balancing (sampling for the learning set or reformulating the problem into the binary question: Is split expected (true) or not (false)?) As of now, the betweenness analysis was still limited to several case studies; in the future, a more rigorous framework would be desired. The idea of analyzing science by the discovery of knowledge changes is general and can be applied to all bibliographical data containing citations. We focused solely on APS journals; however, also papers indexed by PubMed, Web of Science, or Google Scholar may be studied.

## Figures and Tables

**Table 1:**
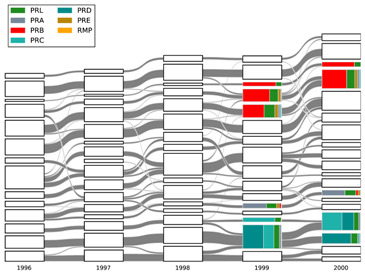
The five evolution events from 1999 to 2000 in the BCN alluvial diagram Figure 4 that we will study quantitatively. The naming convention for TC is that four digits before ‘.’ is the year of TC, two digits after ‘.’ is the position of the TC in the diagram, starting with 00 for the bottom TC; the one just above bottom is 01, and so on. In the left panel, we highlight the related TCs.

TC in 1999	Event	TC in 2000
1999.01	split	2000.02, 2000.03
1999.01, 1999.02	merge	2000.03
1999.04	continue	2000.06
1999.11, 1999.12	merge	2000.15
1999.13	continue	2000.16

**Table 2:** The 25th, 50th, and 75th percentiles of the betweenness of 1849 papers in 1999.01, the 164 papers in 1999.01a, the 17 papers in 1999.01aα, the 147 papers in 1999.01aβ, the 1685 papers in 1999.01b, the 907 papers in 1999.01bα, the 778 papers in 1999.01bβ, the 344 papers in 1999.02, the 144 papers in 1999.02a, the 200 papers in 1999.02b, the 1014 papers in 1999.11, the 299 papers in 1999.11a, the 715 papers in 1999.11b, the 988 papers in 1999.12, the 347 papers in 1999.12a, and the 641 papers in 1999.12b.

	Percentile		
	25	50	75
1999.01	8.06 × 10^−6^	5.73 × 10^−5^	2.05 × 10^−4^
1999.01a	5.90 × 10^−5^	1.58 × 10^−4^	4.67 × 10^−4^
1999.01aα	7.77 × 10^−6^	1.95 × 10^−5^	2.44 × 10^−4^
1999.01aβ	5.29 × 10^−6^	4.96 × 10^−5^	2.48 × 10^−4^
1999.01b	6.22 × 10^−6^	5.04 × 10^−5^	1.88 × 10^−4^
1999.01bα	8.59 × 10^−6^	6.00 × 10^−5^	2.14 × 10^−4^
1999.01bβ	7.97 × 10^−6^	5.32 × 10^−5^	1.83 × 10^−4^
1999.02	2.47 × 10^−6^	5.54 × 10^−5^	2.13 × 10^−4^
1999.02a	3.08 × 10^−5^	1.13 × 10^−4^	3.17 × 10^−4^
1999.02b	2.14 × 10^−7^	1.44 × 10^−5^	1.60 × 10^−4^
1999.11	1.73 × 10^−5^	9.04 × 10^−5^	2.81 × 10^−4^
1999.11a	6.38 × 10^−5^	1.98 × 10^−4^	4.61 × 10^−4^
1999.11b	9.91 × 10^−6^	6.17 × 10^−5^	2.17 × 10^−4^
1999.12	6.56 × 10^−6^	4.54 × 10^−5^	1.62 × 10^−4^
1999.12a	2.74 × 10^−5^	9.08 × 10^−5^	2.33 × 10^−4^
1999.12b	2.52 × 10^−6^	2.69 × 10^−5^	1.20 × 10^−4^

**Table 3:** The distributions of the betweennesses of papers in 1999.04 and 1999.13 that share common references with the other TCs in 1999 (1999.00 to 1999.15). The four columns below 1999.04 and 1999.13 denote the following: the first column shows how many papers have common references with the other TCs, while the second, third, and fourth columns show the lower, median, and upper quartile values of betweennesses of these papers, respectively. For example, there are 25 papers in 1999.04 that share common references with papers in 1999.03, and the betweennesses of these papers have a lower quartile value of 1.6 × 10^−5^, a median value of 4.3 × 10^−4^, and an upper quartile value of 8.1 × 10^−4^. Similarly, there are 254 papers in 1999.13 that share common references with papers in 1999.10, and the betweennesses of these papers have a lower quartile value of 3.6 × 10^−5^, a median value of 8.8 × 10^−5^, and an upper quartile value of 2.7 × 10^−4^. The bottom row “b” represents 1999.04b and 1999.13b, respectively, which are papers in 1999.04 and 1999.13 that have no references in common with papers in other TCs. A betweenness value in red means that it is larger than the maximum of the corresponding quartile distribution of 10^6^ random samples, and a betweenness value in blue denotes it is smaller than the minimum of the corresponding 10^6^ random samples.

	1999.04				1999.13			
	Size	Percentile			Size	Percentile		
		25	50	75		25	50	75
1999.00	12	9.0 × 10^−5^	1.1 × 10^−3^	2.3 × 10^−3^	1	-	-	1.8 × 10^−3^
1999.01	56	1.6 × 10^−4^	4.2 × 10^−4^	1.0 × 10^−3^	6	2.0 × 10^−4^	4.9 × 10^−4^	6.5 × 10^−4^
1999.02	6	3.0 × 10^−4^	5.1 × 10^−4^	7.4 × 10^−4^	2	6.0 × 10^−4^	-	2.6 × 10^−4^
1999.03	25	1.6 × 10^−5^	4.3 × 10^−4^	8.1 × 10^−4^	0	-	-	-
1999.04	-	-	-	-	8	1.5 × 10^−4^	4.8 × 10^−4^	8.0 × 10^−4^
1999.05	179	4.9 × 10^−5^	1.7 × 10^−4^	4.5 × 10^−4^	4	2.2 × 10^−4^	4.3 × 10^−4^	6.5 × 10^−4^
1999.06	110	8.7 × 10^−5^	2.0 × 10^−4^	6.2 × 10^−4^	40	5.9 × 10^−5^	1.6 × 10^−4^	4.5 × 10^−4^
1999.07	29	1.7 × 10^−4^	5.6 × 10^−4^	1.2 × 10^−3^	44	1.4 × 10^−4^	3.1 × 10^−4^	5.5 × 10^−4^
1999.08	63	1.1 × 10^−4^	3.2 × 10^−4^	8.6 × 10^−4^	17	2.2 × 10^−4^	5.2 × 10^−4^	8.5 × 10^−4^
1999.09	49	7.8 × 10^−5^	2.6 × 10^−4^	8.0 × 10^−4^	99	8.0 × 10^−5^	2.5 × 10^−4^	4.8 × 10^−4^
1999.10	53	1.2 × 10^−4^	3.8 × 10^−4^	8.2 × 10^−4^	254	3.6 × 10^−5^	8.8 × 10^−5^	2.7 × 10^−4^
1999.11	89	1.0 × 10^−4^	3.2 × 10^−4^	9.2 × 10^−4^	71	1.4 × 10^−4^	3.4 × 10^−4^	5.7 × 10^−4^
1999.12	53	8.7 × 10^−5^	2.9 × 10^−4^	9.3 × 10^−4^	39	1.3 × 10^−4^	2.7 × 10^−4^	4.6 × 10^−4^
1999.13	9	1.3 × 10^−4^	4.2 × 10^−4^	1.1 × 10^−3^	-	-	-	-
1999.14	62	1.4 × 10^−4^	4.8 × 10^−4^	1.0 × 10^−3^	210	4.2 × 10^−5^	1.0 × 10^−4^	2.7 × 10^−4^
1999.15	17	1.8 × 10^−4^	3.6 × 10^−4^	9.7 × 10^−4^	176	5.1 × 10^−5^	1.3 × 10^−4^	3.1 × 10^−4^
b	88	2.1 × 10^−6^	2.2 × 10^−5^	5.8 × 10^−5^	27	9.1 × 10^−11^	4.3 × 10^−6^	1.8 × 10^−5^

## Supplementary Materials

The following are available online at https://www.mdpi.com/1099-4300/21/12/1152/s1, Figure S1: The scatter plots for simple overlap measure and inclusion measure for CNs between 1981 to 2010, Figure S2: The alluvial diagram of APS papers’ references (CNs) from 1981 to 2010, Figure S3: Bibliographic coupling network in 1991, Table S1: List of all features used in the study.

## Abbreviations

The following abbreviations are used in this manuscript:

PR	Physical Review
PRA	Physical Review A
PRB	Physical Review B
PRC	Physical Review C
PRD	Physical Review D
PRE	Physical Review E
PRL	Physical Review Letters
RMP	Reviews of Modern Physics
BCN	Bibliographic coupling network
CN	Co-citation network
GEP	Group evolution prediction
GED	Group evolution discover
SI	Supplementary Information
