# Supplementary material for: Predicting the Evolution of Physics Research from a Complex Network Perspective
Source: Entropy (Basel). 2019 Nov 26;21(12):1152. doi: 10.3390/e21121152 (PMC7514497; doi:10.3390/e21121152)
Supplement: Supplementary file 1 [file entropy-21-01152-s001.pdf]

Supplementary Information
Predicting the Evolution of Physics Research from a complex network perspective

Wenyuan Liu, Stanisław Saganowski, Przemysław Kazienko, Siew Ann Cheong

The alpha and beta thresholds for event labelling

Two groups in the consecutive time windows are considered similar if at least one of their inclusion measures is greater than alpha or beta parameters. In other words, the alpha and beta parameters are thresholds which have to be satisfied to assign an event between two groups. The theoretical range of values for alpha and beta is between 0% and 100%. However, the most common values are selected from the range from 30% to 70%, depending on the density of the network and node's fluctuation year by year. In general, the selection of parameters should reflect the needs of researchers. For example, one may choose very high value (e.g. 80%) in order to preserve only very similar groups. In another case, it might be necessary to set very low value, e.g. 10% if the network is sparse or the fluctuation is high. In our study, we ran the GED method with alpha and beta parameters varying from 5% to 100%, to see how the number of events varies. Our goal was to have at least one event assigned to each TC. As the splitting and merging events involve several groups, we aimed to have on average slightly more than one event per TC. With this assumption, we selected 30% for both alpha and beta parameters in case of BCN, and 10% for alpha and beta parameters in case of CN. This values produced in total 479 events per 430 groups for BCN, and 492 events per 457 groups for CN.

Correlation between overlap measure and inclusion measure

For BCN, we use the forward and backward intimacy indices to measure the closeness between TCs in consecutive time windows (years). For CN, we considered two types of measure: (i) a simple overlap measure of two groups (the relative fraction of common members), and (ii) an overlap of two groups enriched with the information about the importance of the common members. The latter is suggested by the GED method authors, who named their similarity measure the inclusion measure. One way to evaluate the importance of TC members is to use node centrality measures to rank them within the group. In our work, we are using the Social Position measure[1] (as suggested in the GED method), an idea based on the PageRank algorithm[2]. Saganowski *et al.*[3] found that using a richer similarity measure allows us to track group evolution more reliably. To better understand the difference between the simple overlap measure and the inclusion measure we compared values obtained with both measures in Fig. S1. It turned out that the inclusion measure is on average 20% lower than the simple overlap measure, and the corresponding values, i.e. 30% for the simple overlap and 10% for the inclusion measure, produce roughly the same number of the evolution events. However, the more complex version of the similarity measure (i.e. the inclusion measure), provided slightly better initial prediction results. Therefore, we finally utilized the inclusion measure in our calculations for CN.

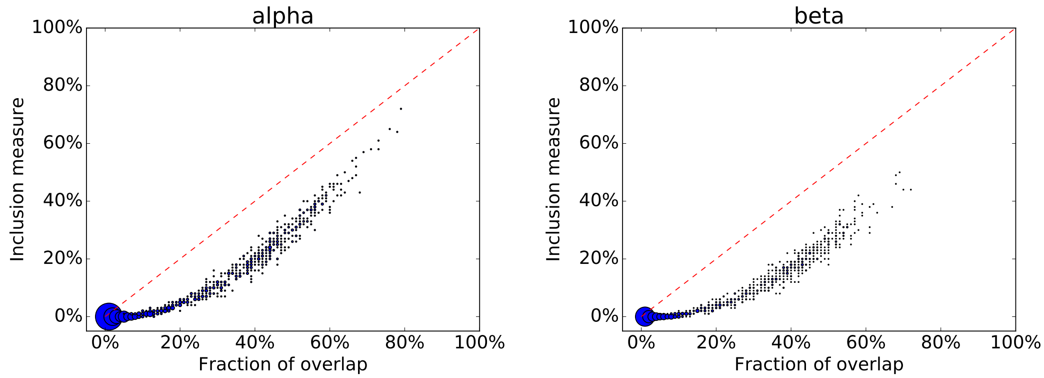

(A) (B)

Figure S1: The scatter plots for simple overlap measure and inclusion measure for CNs between 1981 to 2010. The left panel (A) is for the alpha parameter, i.e. how the groups in t are close to groups in $t+1$. The right panel (B) is for beta parameter, i.e. how the groups in $t+1$ are close to groups in t. The sizes of circles are proportional to the number of instances. The red dash lines are $y = x$ for reference only.

Alluvial diagram for CN

Like the bibliographic coupling network (BCN), the co-citation network (CN) can also be visualized in the form of the alluvial diagram. The groups in a CN represent the papers from the past that are coherent and related to a certain topic that stimulates the present research lines.

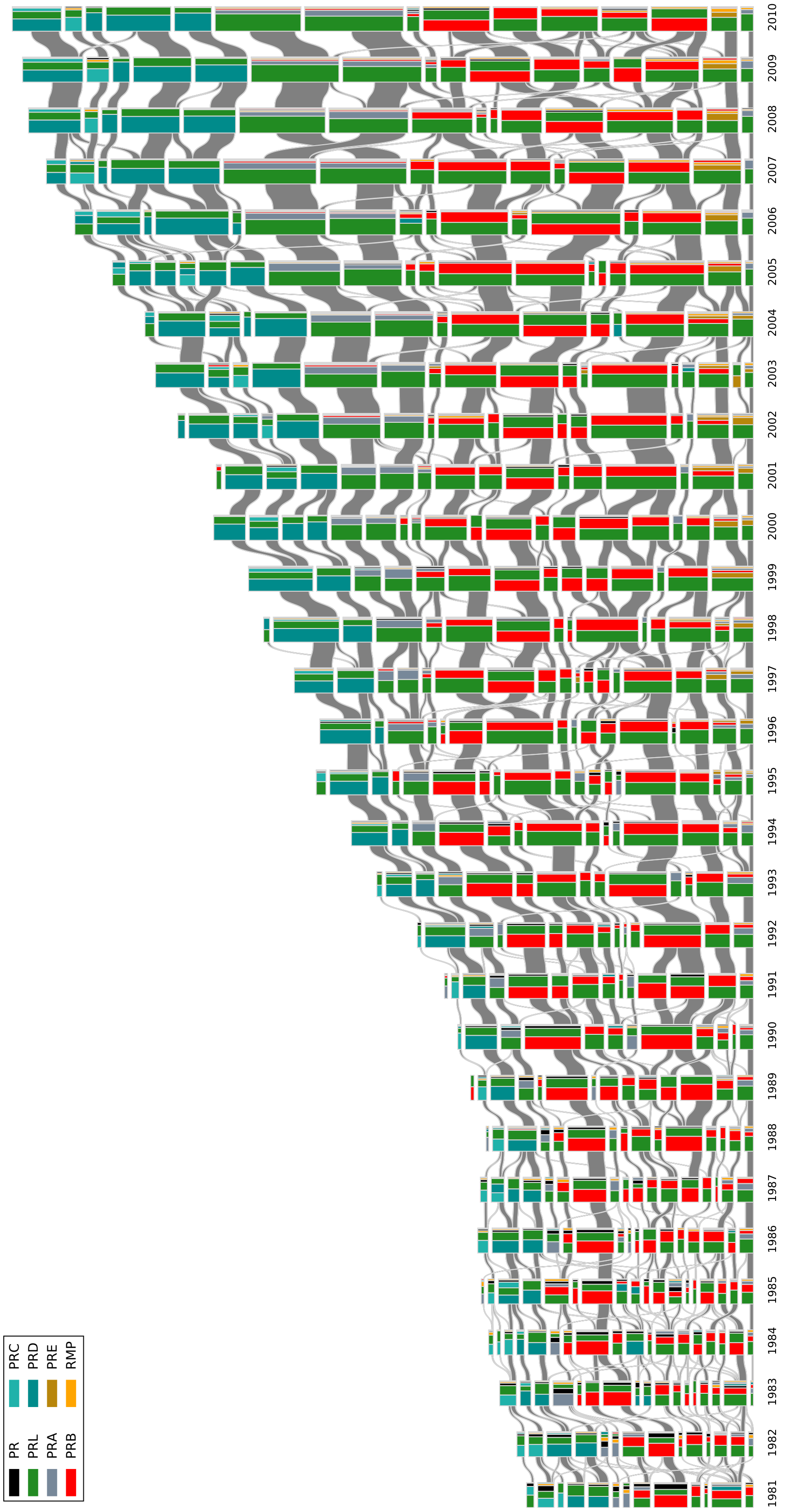

Figure S2: The alluvial diagram of APS papers' references (CNs) from 1981 to 2010. Each block in a column represents a TC extracted from the CN. The height of the block is proportional to the number of papers in the TC. For clarity, only TCs comprising more than 1% of all nodes in CNs are shown. TCs in successive years are connected by streams whose widths at the left and right ends are proportional to the relative overlap percentage. The colours inside a TC represent the relative contributions from different journals.

The list of features used in the study

As we mentioned in **Future events prediction**, each observation contained 77 features (preselected from the initial 100). The full list of 100 features are showed in Tab. S1. Many features in this list are proposed for directed social network, therefore are inappropriate for our undirected BCN and CN. The symbol + indicates this feature was used in BCN prediction, while the symbol ∗ indicates this feature was used in CN prediction.

Table S1: List of all features used in the study. Features proposed in this study are shown in bold.

**Features group**	**Feature name**	**Feature description**
Members/microscopic	sum_group_degree_in	The sum of indegree[4] of nodes belonging to the community calculated within the community. Indegree is a node measure defining the number of connections directed to the node
	avg_group_degree_in	The average value of indegree of nodes belonging to the community calculated within the community
	min_group_degree_in	The minimum value of indegree of nodes belonging to the community calculated within the community
	max_group_degree_in	The maximum value of indegree of nodes belonging to the community calculated within the community
	sum_group_degree_out	The sum of outdegree[4] of nodes belonging to the community calculated within the community. Outdegree is a node measure determining the number of connections outgoing from the node
	avg_group_degree_out	The average value of outdegree of nodes belonging to the community calculated within the community
	min_group_degree_out	The minimum value of outdegree of nodes belonging to the community calculated within the community
	max_group_degree_out	The maximum value of outdegree of nodes belonging to the community calculated within the community
	sum_group_degree_total+∗	The sum of total degree of nodes belonging to the community calculated within the community. Total degree is the sum of indegree and outdegree
	avg_group_degree_total+∗	The average value of total degree of nodes belonging to the community calculated within the community
	min_group_degree_total+∗	The minimum value of total degree of nodes belonging to the community calculated within the community
	max_group_degree_total+∗	The maximum value of total degree of nodes belonging to the community calculated within the community
Continued on next page		

Table S1 – continued from previous page

Features group	Feature name	Feature description
	sum_group_betweenness+∗	The sum of betweenness[4] of nodes belonging to the community calculated within the community. Betweenness is a node measure describing the number of the shortest paths from all nodes to all others that pass through that node
	avg_group_betweenness+∗	The average value of betweenness of nodes belonging to the community calculated within the community
	min_group_betweenness+∗	The minimum value of betweenness of nodes belonging to the community calculated within the community
	max_group_betweenness+∗	The maximum value of betweenness of nodes belonging to the community calculated within the community
	sum_group_closeness+∗	The sum of closeness[4] of nodes belonging to the community calculated within the community. Closeness is a node measure defined as the inverse of the farness, which in turn, is the sum of distances to all other nodes
	avg_group_closeness+∗	The average value of closeness of nodes belonging to the community calculated within the community
	min_group_closeness+∗	The minimum value of c of nodes belonging to the community calculated within the community
	max_group_closeness+∗	The maximum value of closeness of nodes belonging to the community calculated within the community
	sum_group_eigenvector+∗	The sum of eigenvector[5] of nodes belonging to the community calculated within the community. Eigenvector is a node measure indicating the influence of a node in the network
	avg_group_eigenvector+∗	The average value of eigenvector of nodes belonging to the community calculated within the community
	min_group_eigenvector+∗	The minimum value of eigenvector of nodes belonging to the community calculated within the community
	max_group_eigenvector+∗	The maximum value of eigenvector of nodes belonging to the community calculated within the community
	avg_group_eccentricity+∗	The average value of eccentricity[6] of nodes belonging to the community calculated within the community. Eccentricity of a node is its shortest path distance from the farthest other node in the graph

Continued on next page

Table S1 – continued from previous page

Features group	Feature name	Feature description
	min_group_eccentricity+*	The minimum value of eccentricity of nodes belonging to the community calculated within the community
	max_group_eccentricity+*	The maximum value of eccentricity of nodes belonging to the community calculated within the community
	sum_network_degree_in	The sum of indegree of nodes belonging to the community calculated within the network
	avg_network_degree_in	The average value of indegree of nodes belonging to the community calculated within the network
	min_network_degree_in	The minimum value of indegree of nodes belonging to the community calculated within the network
	max_network_degree_in	The maximum value of indegree of nodes belonging to the community calculated within the network
	sum_network_degree_out	The sum of outdegree of nodes belonging to the community calculated within the network
	avg_network_degree_out	The average value of outdegree of nodes belonging to the community calculated within the network
	min_network_degree_out	The minimum value of outdegree of nodes belonging to the community calculated within the network
	max_network_degree_out	The maximum value of outdegree of nodes belonging to the community calculated within the network
	sum_network_degree_total+*	The sum of total degree of nodes belonging to the community calculated within the network
	avg_network_degree_total+*	The average value of total degree of nodes belonging to the community calculated within the network
	min_network_degree_total+*	The minimum value of total degree of nodes belonging to the community calculated within the network
	max_network_degree_total+*	The maximum value of total degree of nodes belonging to the community calculated within the network
	sum_network_betweenness +*	The sum of betweenness of nodes belonging to the community calculated within the network
	avg_network_betweenness+*	The average value of betweenness of nodes belonging to the community calculated within the network

Continued on next page

Table S1 – continued from previous page

Features group	Feature name	Feature description
	min_network_betweenness+∗	The minimum value of betweenness of nodes belonging to the community calculated within the network
	max_network_betweenness+∗	The maximum value of betweenness of nodes belonging to the community calculated within the network
	sum_network_closeness+∗	The sum of closeness of nodes belonging to the community calculated within the network
	avg_network_closeness+∗	The average value of closeness of nodes belonging to the community calculated within the network
	min_network_closeness+∗	The minimum value of closeness of nodes belonging to the community calculated within the network
	max_network_closeness+∗	The maximum value of closeness of nodes belonging to the community calculated within the network
	sum_network_eigenvector+∗	The sum of eigenvector of nodes belonging to the community calculated within the network
	avg_network_eigenvector+∗	The average value of eigenvector of nodes belonging to the community calculated within the network
	min_network_eigenvector+∗	The minimum value of eigenvector of nodes belonging to the community calculated within the network
	max_network_eigenvector+∗	The maximum value of eigenvector of nodes belonging to the community calculated within the network
	avg_group_coefficient[7]+∗	The average of the local clustering coefficients of all the nodes in the community
	avg_network_coefficient[7]+∗	The average of the local clustering coefficients of all the nodes in the network
Group/mesoscopic	group_size+∗	The number of nodes in the group
	group_density[7]+∗	The number of connections between nodes in the group in relation to all possible connections between them
	group_cohesion[8]+∗	The vertex connectivity of the community
	group_coefficient_global[7]+∗	The ratio of the triangles and the connected triples in the community
	group_reciprocity[9]	A fraction of edges that are reciprocated within the community
	group_leadership[4]+∗	A measure describing centralization in the community (the largest value is for a star network)

Continued on next page

Table S1 – continued from previous page

Features group	Feature name	Feature description
	neighborhood_out	The number of nodes outside the community that have incoming connection from the nodes inside the community divided by the number of nodes in the community
	neighborhood_in	The number of nodes outside the community that have outgoing connection to the nodes inside the community divided by the number of nodes in the community
	neighborhood_all+∗	The number of nodes outside the community that are connected to the nodes inside the community divided by the number of nodes in the community
	group_adhesion[8]+∗	The minimum number of edges needed to be removed to obtain a community which is not strongly connected
	alpha[10]	The GED inclusion measure of group G_i from time window T_n in group G_j from T_{n+1}
	beta[10]	The GED inclusion measure of group G_j from time window T_{n+1} in group G_i from T_n
	network_ratio_size+∗	The ratio of *group_size* to *network_size*
	network_ratio_density+∗	The ratio of *group_density* to *network_density*
	network_ratio_cohesion+∗	The ratio of *group_cohesion* to *network_cohesion*
	network_ratio_coefficient_global+∗	The ratio of *group_coefficient_global* to *network_coefficient_global*
	network_ratio_coefficient_average+∗	The ratio of *group_clustering_coefficient* to *network_clustering_coefficient*
	network_ratio_reciprocity	The ratio of *group_reciprocity* to *network_reciprocity*
	network_ratio_leadership+∗	The ratio of *group_leadership* to *network_leadership*
	network_ratio_eccentricity+∗	The ratio of *avg_group_eccentricity* to *network_avg_eccentricity*
	network_ratio_adhesion+∗	The ratio of *group_adhesion* to *network_adhesion*
	**phys_rev**∗	**The number of articles belonging to the group that were published in the Physical Review journal**
	**phys_rev_a**+∗	**The number of articles belonging to the group that were published in the Physical Review A journal**
	**phys_rev_b**+∗	**The number of articles belonging to the group that were published in the Physical Review B journal**
	**phys_rev_c**+∗	**The number of articles belonging to the group that were published in the Physical Review C journal**

Continued on next page

Table S1 – continued from previous page

**Features group**	**Feature name**	**Feature description**
	**phys_rev_d+∗**	**The number of articles belonging to the group that were published in the Physical Review D journal**
	**phys_rev_e+∗**	**The number of articles belonging to the group that were published in the Physical Review E journal**
	**phys_rev_lett+∗**	**The number of articles belonging to the group that were published in the Physical Review Letters journal**
	**phys_rev_stab+∗**	**The number of articles belonging to the group that were published in the Physical Review STAB journal**
	**phys_rev_stper+**	**The number of articles belonging to the group that were published in the Physical Review STPER journal**
	**physics∗**	**The number of articles belonging to the group that were published in the Physics journal**
	**rev_mod_phys+∗**	**The number of articles belonging to the group that were published in the Review of Modern Physics journal**
	**sum_group_age+∗**	**The sum of age of articles belonging to the group. In the co-reference network the age of an article is the average age of the articles it references to. In the co-citation network the age of an article is the age of the articles being cited.**
	**avg_group_age+∗**	**The average age of articles belonging to the group**
	**min_group_age+∗**	**The minimum age of articles belonging to the group**
	**max_group_age+∗**	**The maximum age of articles belonging to the group**
	**network_ratio_avg_group_age+∗**	**The ratio of avg_group_age to the average age of all articles in the network**
	time_window+∗	The number of time window from which the community instance was obtained
Network/macroscopic	network_size+∗	The number of nodes in the network
	network_density+∗	The number of connections between nodes in the network in relation to all possible connections between them
	network_cohesion+∗	The vertex connectivity of the network
	network_coefficient_global+∗	The ratio of the triangles and the connected triples in the network
	network_coefficient_average+∗	The average of the local clustering coefficients of all the nodes in the network

Continued on next page

Table S1 – continued from previous page

Features group	Feature name	Feature description
	network_reciprocity	A fraction of edges that are reciprocated within the network
	network_leadership+∗	A measure describing centralization in the network (the largest value is for a star network)
	network_avg_eccentricity+∗	The average value of eccentricity of nodes within the network.
	network_adhesion+∗	The minimum number of edges needed to be removed to obtain a graph which is not strongly connected

The visualization of TC

In Fig. S3 we show the topic clusters for BCN in 1991. By checking top cited papers in each TC, we found the red TC (top) is mainly about *ab initio* calculation; the orange TC (top right) is mainly about high temperature superconductor; the dark blue TC is mainly about quantum dot and quantum well; the black TC is mainly about excitonic effect in quantum well; the green TC is mainly about electromagnetic related optics; the light blue TC is mainly about surface physics and statistical physics; the purple TC is mainly about cosmology; the yellow TC is mainly about particle physics. The above descriptions are not rigorous measurements, but subjective impressions based on author's knowledge about physics. The intention is to give readers a sensing of TC space rather than a quantitative study.

References

[1] Brodka, P., Musial, K. & Kazienko, P. A Performance of Centrality Calculation in Social Networks. In *CASoN - International Conference on Computational Aspects of Social Networks*, 24–31 (IEEE, 2009). DOI 10.1109/CASoN.2009.20.

[2] Page, L., Brin, S., Motwani, R. & Winograd, T. The PageRank Citation Ranking: Bringing Order to the Web. Tech. Rep. (1999).

[3] Saganowski, S., Bródka, P. & Kazienko, P. Influence of the User Importance Measure on the Group Evolution Discovery. *Foundations of Computing and Decision Sciences* **37** (2012). DOI 10.2478/v10209-011-0017-6.

[4] Freeman, L. C. Centrality in social networks conceptual clarification. *Social Networks* **1**, 215–239 (1978). DOI 10.1016/0378-8733(78)90021-7.

[5] Bonacich, P. Factoring and weighting approaches to status scores and clique identification. *The Journal of Mathematical Sociology* **2**, 113–120 (1972). DOI 10.1080/0022250X.1972.9989806.

[6] Harary, F. *Graph theory* (Addison-Wesley, Reading, MA, 1969).

[7] Wasserman, S. & Faust, K. *Social Network Analysis: Methods and Applications* (Cambridge University Press, Cambridge, 1994). DOI 10.1017/CBO9780511815478.

[8] White, D. R. & Harary, F. The Cohesiveness of Blocks In Social Networks: Node Connectivity and Conditional Density. *Sociological Methodology* **31**, 305–359 (2001). DOI 10.1111/0081-1750.00098.

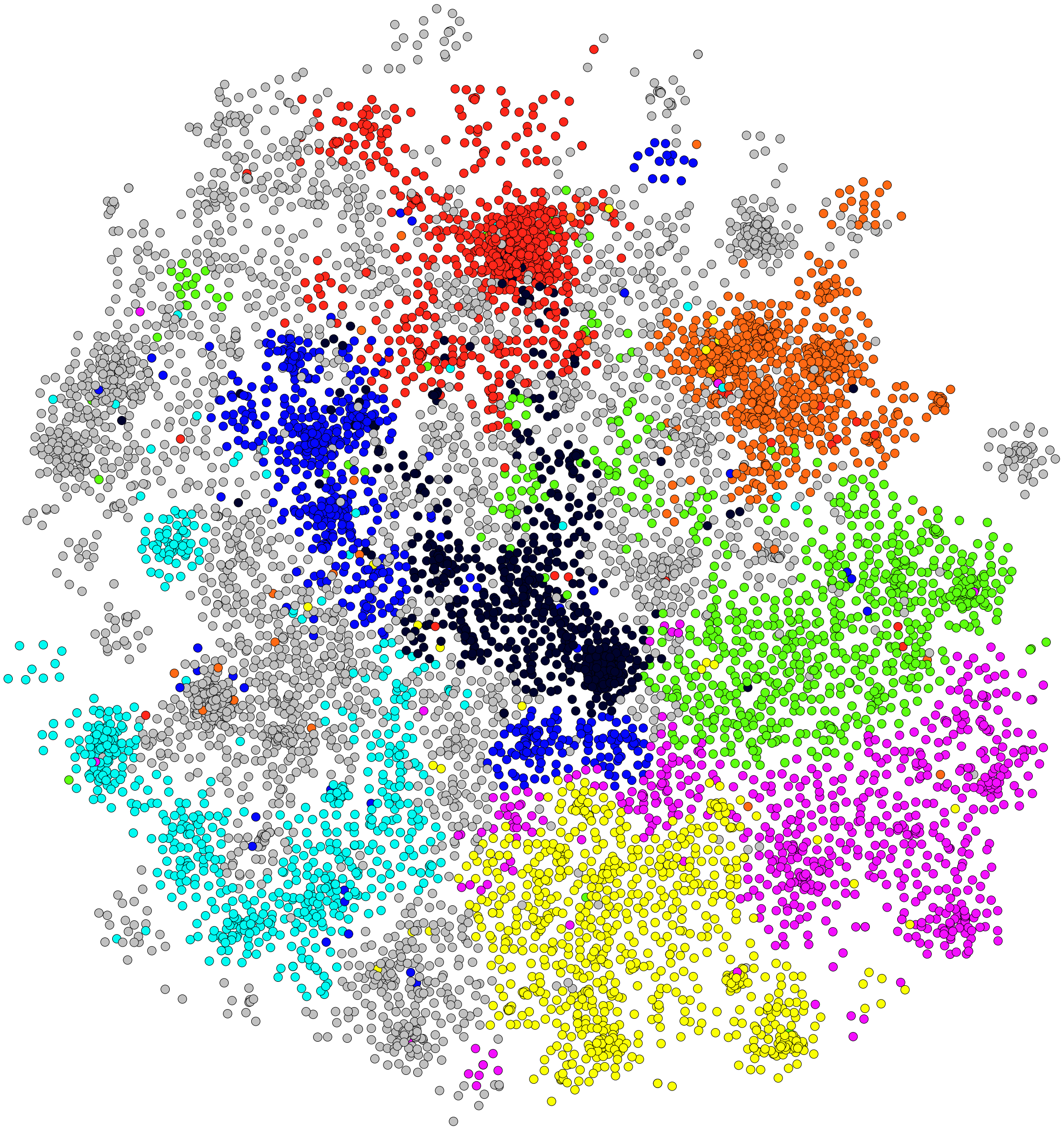

Figure S3: Bibliographic coupling network in 1991. Each node represents a paper. Papers in the same community (detected by Louvain method) are given the same color for eight largest communities, papers in other smaller communities are grey. The layout algorithm be used is “OpenOrd” in Gephi.

[9] Newman, M. *Networks: An Introduction* (Oxford University Press, 2010). DOI 10.1093/acprof:oso/9780199206650.001.0001.

[10] Bródka, P., Saganowski, S. & Kazienko, P. GED: the method for group evolution discovery in social networks. *Social Network Analysis and Mining* **3**, 1–14 (2013). DOI 10.1007/s13278-012-0058-8.
